# Mutation of lipoprotein processing pathway gene *lspA* or inhibition of LspA activity by globomycin increases MRSA resistance to β-lactam antibiotics

**DOI:** 10.1128/aac.01276-25

**Published:** 2025-12-29

**Authors:** Claire Fingleton, Merve S. Zeden, Emilio Bueno, Felipe Cava, James P. O'Gara

**Affiliations:** 1 Microbiology, School of Natural Sciences, National University of Ireland 8799, Galway, Ireland; 2 Department of Molecular Biology, Umeå University, MIMS - Laboratory for Molecular Infection Medicine Sweden 8075, Umeå, Sweden; The Peter Doherty Institute for Infection and Immunity, Melbourne, Victoria, Australia

**Keywords:** resistance, lipoprotein, globomycin, MRSA, beta-lactam

## Abstract

Resistance to β-lactam antibiotics in methicillin-resistant *Staphylococcus aureus* is mediated by the *mecA*-encoded, β-lactam-resistant transpeptidase, penicillin-binding protein 2a (PBP2a), which is capable of crosslinking peptidoglycan in the presence of β-lactam antibiotics. Here, we report that mutation of the lipoprotein signal peptidase II gene, *lspA*, from the lipoprotein processing pathway, significantly increased β-lactam resistance in MRSA, independent of changes in PBP2a levels or peptidoglycan composition. Exposure of MRSA to the LspA inhibitor globomycin also increased β-lactam resistance. Mutation of *lgt*, which encodes diacylglycerol transferase (Lgt) responsible for synthesis of the LspA substrate, did not impact β-lactam susceptibility. Furthermore, mutation of *lgt* in an *lspA* background restored β-lactam resistance to wild-type levels. These data suggest that accumulation of the LspA substrate, diacylglyceryl-lipoprotein, is associated with increased β-lactam resistance in MRSA.

## INTRODUCTION

The cell envelope of *Staphylococcus aureus* comprises a cytoplasmic membrane surrounded by a thick peptidoglycan layer, cell wall-anchored proteins, lipoteichoic acids (LTA), wall teichoic acids (WTA), and cell surface proteins. Accurate biosynthesis, assembly, and stability of these cell envelope components is essential for the growth and pathogenesis of *S. aureus* and is the target of numerous antimicrobial agents (1). Methicillin resistance in methicillin-resistant *S. aureus* (MRSA) is mediated by the *mecA*-encoded, low-affinity penicillin-binding protein 2a (PBP2a) carried on the mobile staphylococcal cassette chromosome *mec* (SCC*mec*). Carriage of the SCC*mec* element by *S. aureus* is associated with the expression of heterogenous, low-level resistance (HeR) to β-lactam antibiotics (2–4). Growth of HeR MRSA strains on elevated β-lactam concentrations can select for homogeneously resistant (HoR) MRSA mutants. Many accessory mutations associated with the HoR phenotype occur in chromosomal loci outside SCC*mec* and are frequently associated with the activation of the stringent response and cyclic-di-adenosine monophosphate (c-di-AMP) signaling pathways (5–10), the activity of RNA polymerase (11), and the ClpXP chaperone-protease complex (12, 13). In addition, methicillin susceptible *S. aureus* strains (MSSA) lacking *mecA* can also acquire low-level resistance through adaptive mutations impacting the c-di-AMP signaling pathway and ClpXP activity (14).

Bacterial lipoproteins are a class of lipid-modified membrane proteins, involved in a range of diverse functions such as nutrient acquisition (15), signal transduction (16), respiration (17), protein folding (18), virulence (19), antibiotic resistance (20), and host invasion (21). Mature lipoproteins are composed of lipid moieties, specifically acyl groups, linked to the N-terminus of a protein. The hydrophobic nature of the acyl groups serves as a membrane anchor for the lipoprotein (22). In Gram-negative bacteria, lipoproteins reside in both the cytoplasmic and outer membranes, while in Gram-positive bacteria, they are anchored in the outer leaflet of the cytoplasmic membrane and the protein portion may extend into the cell wall and beyond (23).

Lipoprotein genes are estimated to comprise 1%–3% of all genes in bacterial genomes (23). While many lipoproteins have been identified and experimentally validated, others have been identified only using predictive software, and their functions remain unknown (24). A recent bioinformatic evaluation of staphylococcal lipoproteins in the MRSA USA300 identified 67 lipoproteins, comprising 2.57% of all genes (15, 23). When grouped by function, 25 of the 67 lipoproteins were implicated in ion (notably iron) and nutrient transport, 8 were ascribed miscellaneous functions including sex pheromone biosynthesis, respiration, chaperone-folding, and protein translocation, and 15 were classified as tandem lipoproteins, of which 9 are “lipoprotein-like” lipoproteins, known to play a role in host cell invasion (21). The remaining 19 lipoproteins were not assigned any known function.

Lipoproteins are synthesized in a precursor form called preprolipoproteins. The N-terminal domain includes a type II signal peptide, approximately 20 amino acids in length (25), which enables translocation of preprolipoproteins to the cytoplasmic membrane, predominantly via the general secretory (Sec) pathway (26). The signal peptide has three distinct domains: a positively charged N domain, a hydrophobic H domain, and a C-terminal lipobox. The lipobox is comprised of a conserved 3-amino acid sequence [LVI]_−3_ [ASTVI]_−2_ [GAS]_−1_ in front of an invariant cysteine residue [C]_+1_ (27, 28). This lipobox serves as a recognition site for enzymes of the lipoprotein processing pathway, enabling lipid modification of the cysteine residue and cleavage of the signal peptide between the amino acid at position −1 and the +1 cysteine (27).

The first enzyme in the lipoprotein processing pathway is diacylglycerol transferase (Lgt) which covalently attaches a diacylglycerol molecule from phosphatidyl glycerol onto the sulfhydryl group of the invariant cysteine, resulting in a prolipoprotein (29). This diacylglycerol serves as a membrane anchor. Next, the type II lipoprotein signal peptidase (Lsp) cleaves the signal peptide between the amino acid at position −1 and +1, leaving the invariant cysteine residue as the new terminal amino acid (30). Lgt and Lsp are conserved in all bacterial species. In Gram-negative bacteria, a third step is catalyzed by the enzyme N-acyl transferase (Lnt), which transfers an N-acyl group onto the invariant cysteine residue at the N-terminal of the protein (31). Lnt homologs have been identified in high-GC Gram-positive bacteria (32) but not in low-GC Firmicutes. Despite the lack of an apparent Lnt homolog, N-acylated lipoproteins have been identified in *S. aureus* (33), and recent work has identified two novel non-contiguous genes *lnsA* and *lnsB* which catalyze the N-terminal acylation of lipoproteins in *S. aureus* (34). The membrane metalloprotease Eep and the EcsAB transporter were shown to be involved in the processing and export of linear peptides, including the signal peptide cleaved by LspA in the lipoprotein processing pathway (35–37). Lgt and LspA are conditionally essential for the viability of Gram-negative *Salmonella Typhimurium* (38). In contrast, *lgt* and *lspA* mutations do not impact viability in Gram-positive *Bacillus subtilis* (39) but are associated with changes in growth, immunogenicity (40), and virulence (19) phenotypes.

In this study, we characterized the impact of *lspA and lgt* mutations, alone and in combination, on susceptibility to β-lactam antibiotics, growth, PBP2a expression, peptidoglycan structure, and autolytic activity in MRSA. The impact of globomycin, which is known to inhibit LspA activity, on β-lactam susceptibility was also characterized. Our data suggest that accumulation of the LspA substrate, diacylglyceryl-prolipoprotein, modulates resistance to β-lactam antibiotics in MRSA.

## RESULTS

### Mutation of *lspA* in MRSA increases resistance to β-lactam antibiotics

**Fig 1:**
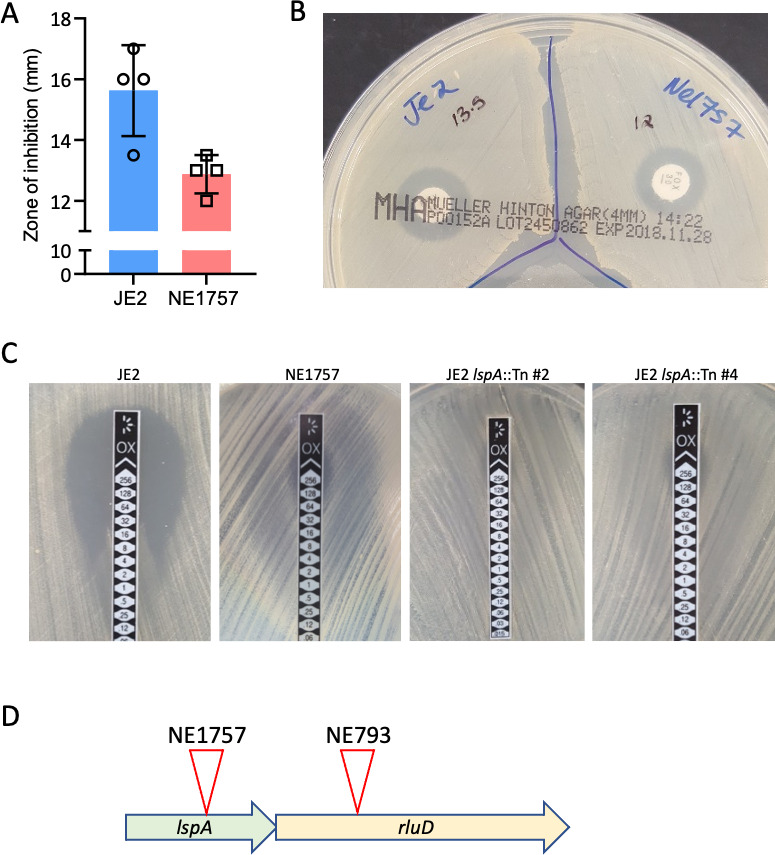
Mutation of *lspA* in MRSA increases resistance to cefoxitin and oxacillin. (**A**) Average diameters of the cefoxitin disk zones of inhibition for JE2 and NE1757 (*lspA*::Tn) from four independent experiments, plotted using Prism software (GraphPad). (**B**) Representative image of JE2 (left) and NE1757 (right) grown on MH agar with a cefoxitin 30 μg disk. (**C**) M.I.C.Evaluator measurement of oxacillin minimum inhibitory concentrations (MICs) in JE2, NE1757 (*lspA*::Tn), and two independent JE2 transductants (#2 and #4) carrying the *lspA*::Tn allele grown on MHB 2% NaCl agar. This assay was repeated three independent times for each strain, and a representative image is shown. (**D**) Organization of the *lspA*/*rluD* operon including the approximate locations of the transposon insertions in NE1757 and NE793.

The Nebraska Transposon Mutant Library (NTML) (41) was screened to identify mutants exhibiting altered susceptibility to cefoxitin, which is recommended as a surrogate for measuring *mecA*-mediated oxacillin resistance in clinical laboratories (42) in accordance with Clinical and Laboratory Standards Institute (CLSI) guidelines for disk diffusion susceptibility assays. Mutants identified by this screen included NE869 (*yjbH*::Tn) (43), NE1909 (*sagA*::Tn) (44), and NE810 (*cycA*::Tn) (45), all of which have previously been implicated in β-lactam resistance. A new mutant identified in this screen was NE1757 (*lspA*::Tn), which exhibited increased resistance to cefoxitin (Fig. 1A and B). Using E-test strips, the oxacillin MIC of the *lspA* transposon mutant NE1757 was found to be 128–256 μg/mL, compared to 32–64 μg/mL for JE2 (Fig. 1C). Two randomly selected JE2 transductants carrying the *lspA*::Tn allele from NE1757 also exhibited increased resistance to oxacillin (Fig. 1C). The *lspA* gene appears to be in a 2-gene operon with the pseudouridylate synthase gene, *rluD* (Fig. 1D), which was recently implicated in resistance to tetracycline (46). The oxacillin MIC of the NTML *rluD*::Tn mutant NE793 was that same as JE2 (32 µg/mL), indicating that the increased β-lactam resistance of NE1757 was not due to downstream polar effects of the transposon insertion in *lspA*.

The increased oxacillin resistance phenotype of NE1757 was complemented by the introduction of a plasmid (pLI50)-borne copy of the wild-type *lspA* gene (p*lspA*) into the mutant (Table 1; Fig. S1). Comparison of JE2 and NE1757 growth in MHB, MHB 2% NaCl, and TSB revealed no significant differences (Fig. S2). Similarly, population analysis profiling revealed that the heterogeneous pattern of oxacillin resistance expressed by JE2 was unchanged in NE1757 (Fig. S3). These observations indicate that the increased β-lactam resistance phenotype of NE1757 was not attributable to any growth advantage or change in the heterogeneous/homogeneous oxacillin resistance profile.

**TABLE 1:** Antibacterial activity of oxacillin, cefotaxime, nafcillin, vancomycin (MIC measurements; µg/mL^*a*^) against ATCC 29213 (control), JE2, NE1757 (*lspA*::Tn), NE1757 p*lspA*, NE1757 pLI50, JE2 *lspA*::Tn #2 (transductant), NE1905 (*lgt*::Tn), NE1757/NE1905 double mutant *lspA*/*lgt* and NE107 (*ecsB*)

Strain	Oxacillin	Cefotaxime	Nafcillin	Vancomycin
ATCC 29213	≥1	2	0.5	1
JE2	32–64	64	32	1
NE1757 (*lspA*)	128–256	256	64	1
NE1757 p*lspA*	64	64	32	1
NE1757 pLI50	256	256	–^*b*^	1
JE2 *lspA*::Tn #2	128–256	256	–	1
NE1905 (*lgt*)	64	64	32	1
*lspA* MM/NE1905 (*lspA*/*lgt*)	64	128	32	1
NE107 (*ecsB*)	64	64	32	1

^*a*^ MIC measurements (µg/mL) were performed by MH agar dilution in accordance with CLSI standards.

^*b*^ "–", not determined.

Comparative WGS analysis confirmed that the only change in the NE1757 genome was the insertion of the *Bursa aurealis* transposon in the *lspA* gene, and there were no single nucleotide polymorphisms (SNPs) present. The NE1757 genome was also checked manually for zero coverage regions to confirm the absence of any large deletions and insertions.

### Mutation of *lspA* does not affect PBP2a expression or peptidoglycan structure and crosslinking

**Fig 2:**
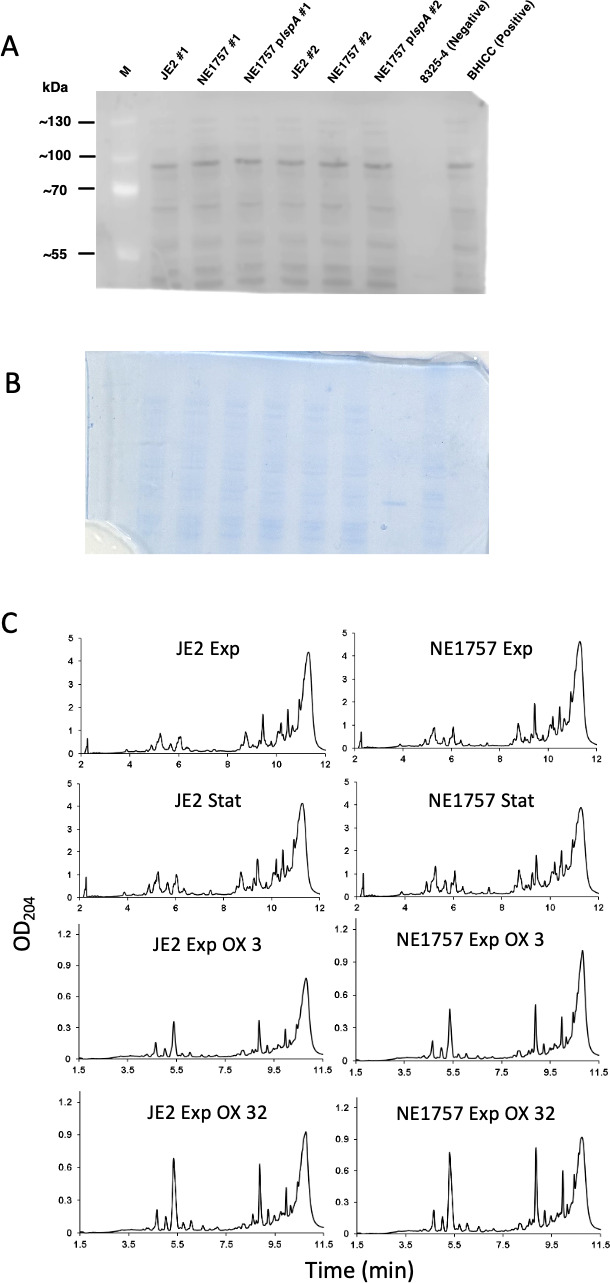
Mutation of *lspA* does not affect PBP2a expression levels or peptidoglycan structure and crosslinking. (**A**) Western blot of PBP2a protein in JE2, NE1757 (*lspA*), NE1757 p*lspA,* MSSA strain 8325-4 (negative control), and MRSA strain BHICC (positive control). Cells were grown to exponential stage in MHB 2% NaCl supplemented with 0.5 µg/mL oxacillin, with the exception of 8325-4 which was grown in MHB 2% NaCl. For each sample, 8 µg total protein was run on a 7.5% Tris-Glycine gel, transferred to a PVDF membrane and probed with anti-PBP2a (1:1,000), followed by HRP-conjugated protein G (1:2,000) and chemiluminescence detection with the Amersham ECL Western Blotting Detection kit. The blot was imaged using a LICOR C-Digit Blot scanner using the Image Studio Software (version 6.1). Four independent experiments were performed, and a representative image is shown. (**B**) Coomassie staining of the Tris-Glycine gel after transfer of protein to the PVDF membrane used in the PBP2a Western blot. (**C**) Representative UV chromatograms of peptidoglycan extracted from JE2 and NE1757 collected from cultures grown to exponential (Exp) or stationary (Stat) phase in MHB or MHB supplemented with oxacillin (OX) 3 µg/mL or 32 µg/mL. Each profile shown is a representative of three biological replicates.

Western blotting was used to compare PBP2a levels in JE2, NE1757, and NE1757 p*lspA*. The MSSA strain 8325-4 was included as a *mecA*-negative control. Growth of JE2 and NE1757 in MHB without 2% NaCl at 37°C (data not shown) or MHB with 2% NaCl at 35°C supplemented with 0.5 µg/mL oxacillin (Fig. 2A and B) revealed similar levels of PBP2a expression in all strains.

Quantitative peptidoglycan compositional analysis was performed on muramidase-digested muropeptide fragments extracted from exponential or stationary phase cultures of JE2 and NE1757 grown in MHB or MHB supplemented with oxacillin 3 µg/mL or 32 µg/mL. The PG profile of JE2 and the *lspA* transposon mutant NE1757 were similar under all growth conditions tested (Fig. 2C). Thus, supplementation of MHB with oxacillin was associated with significant changes in muropeptide oligomerization and reduced crosslinking, but these effects were the same in both JE2 and NE1757 (Fig. S4). The total PG concentrations extracted from JE2 and NE1757 cell pellets were also the same. Consistent with the absence of any changes in PG architecture, Triton X-100-induced autolysis profiles for JE2 and NE1757 were identical (Fig. S5).

### Exposure to the LspA inhibitor globomycin also increases β-lactam resistance

Globomycin is a natural peptide antibiotic, first discovered in 1978 (47–49) that inhibits LspA by sterically blocking the active site of the enzyme (49). Globomycin has moderate to strong antibacterial activity against many Gram-negative species and has been proposed to cause disruption of cell surface integrity (50). However, despite its ability to inhibit LspA, globomycin does not have significant antimicrobial activity against Gram-positive bacteria including *S. aureus*, with MICs >100 µg/mL (47, 48, 51).

**Fig 3:**
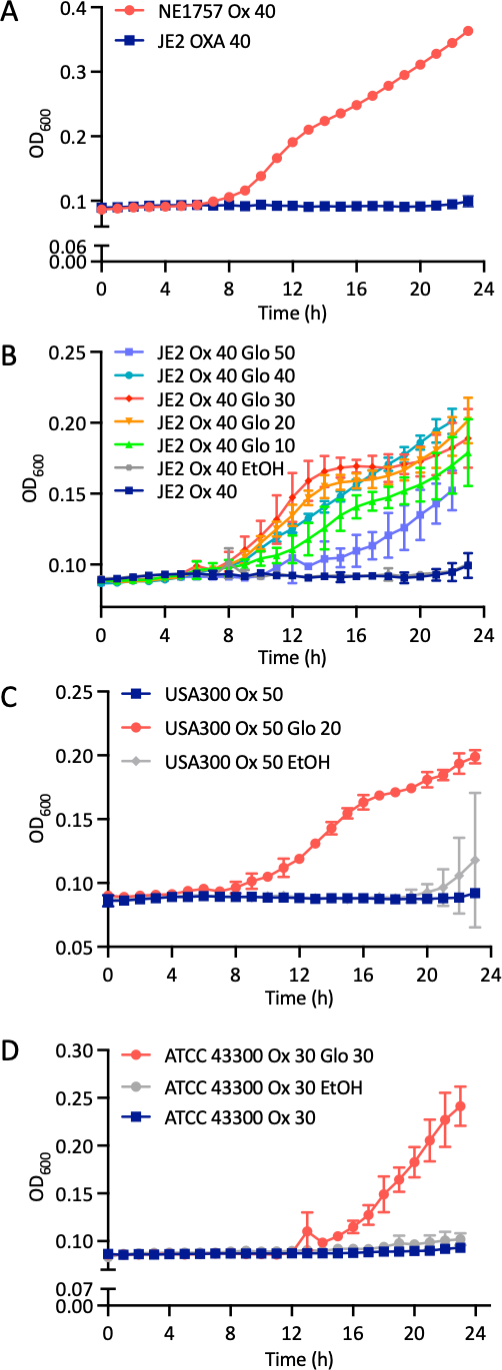
Mutation of *lspA* or exposure to globomycin increases oxacillin resistance. (**A**) JE2 and NE1757 (*lspA*) grown in MHB 2% NaCl supplemented with oxacillin (Ox) 40 µg/mL. (**B**) JE2 grown in MHB 2% NaCl supplemented with oxacillin 40 µg/mL and globomycin (Glo) concentrations ranging from 10 to 50 µg/mL or MHB 2% NaCl supplemented with oxacillin 40 µg/mL and 0.6% ethanol (control solvent for globomycin). (**C**) FPR3757 grown in MHB 2% NaCl supplemented with oxacillin 50 µg/mL, MHB 2% NaCl supplemented with oxacillin 50 µg/mL and globomycin 20 µg/mL, or MHB 2% NaCl supplemented with oxacillin 50 µg/mL and 0.6% ethanol (control solvent for globomycin). (**D**) ATCC 43300 grown in MHB 2% NaCl supplemented with oxacillin 30 µg/mL, MHB 2% NaCl supplemented with oxacillin 30 µg/mL and globomycin 30 µg/mL, or MHB 2% NaCl supplemented with oxacillin 50 µg/mL and 0.6% ethanol (EtOH, control solvent for globomycin). The oxacillin and globomycin concentrations used in these experiments were determined empirically for each strain. Cultures were grown in a Tecan Sunrise incubated microplate reader for 24 h at 35°C. OD_600_ was recorded at 1 h intervals, and growth curves were plotted in Prism software (GraphPad). The data presented are the average of three independent biological replicates, and error bars represent standard deviations.

Because the *lspA*::Tn allele was associated with increased resistance to β-lactams, we hypothesized that chemical inhibition of LspA by globomycin may also be associated with increased β-lactam resistance. To test this hypothesis, the susceptibility of JE2 and NE1757 to oxacillin was determined in the presence or absence of globomycin. As predicted, oxacillin 40 µg/mL inhibited the growth of JE2 but not NE1757 (Fig. 3A). Next, JE2 MHB 2% NaCl 40 µg/mL oxacillin cultures were further supplemented with 10, 20, 30, 40, or 50 µg/mL globomycin (Fig. 3B). Oxacillin-induced inhibition of JE2 growth was rescued by globomycin 10, 20, and 30 µg/mL (Fig. 3B). Growth of JE2 in 40 µg/mL oxacillin and 50 µg/mL globomycin was substantially impacted compared to lower globomycin concentration (Fig. 3B) indicating that the antagonism of oxacillin by globomycin was dose dependent.

These experiments were extended to FPR3757 (from the USA300 lineage) and ATCC43300, a SCC*mec* type II MRSA clinical isolate. Oxacillin concentrations of 50 µg/mL and 30 µg/mL inhibited growth of FPR3757 and ATCC4330, respectively (Fig. 3C and D). Globomycin concentrations (determined empirically for each strain) of 20 µg/mL for FPR3757 (Fig. 3C) and 30 µg/mL for ATCC43300 (Fig. 3D) rescued growth in the presence of oxacillin. These data demonstrate that the increased oxacillin resistance phenotype observed in the *lspA* mutant NE1757 can be replicated by globomycin-induced inhibition of LspA activity in wild-type JE2 and other MRSA strains.

In contrast to the observation that globomycin increased β-lactam resistance in JE2, and two additional MRSA strains, the growth of NE1757 in a range of globomycin concentrations from 10 to 50 µg/mL had a dose-dependent and negative effect on growth in the presence of oxacillin 40 µg/mL (Fig. S6A). Furthermore, JE2 and NE1757 exhibited similar susceptibility to globomycin alone (at concentrations of 10–50 μg/mL) (Fig. 6B and C). Taken together, these data suggest that globomycin antagonizes β-lactam antibiotics, increasing MRSA resistance to oxacillin and cefotaxime in a LspA-dependent manner. However, in the *lspA* transposon mutant, the combination of globomycin and oxacillin interferes with the growth of JE2, particularly at higher concentrations of globomycin, perhaps due to off-target effects or an unidentified phenotypic vulnerability associated with the absence of LspA.

**Fig 4:**
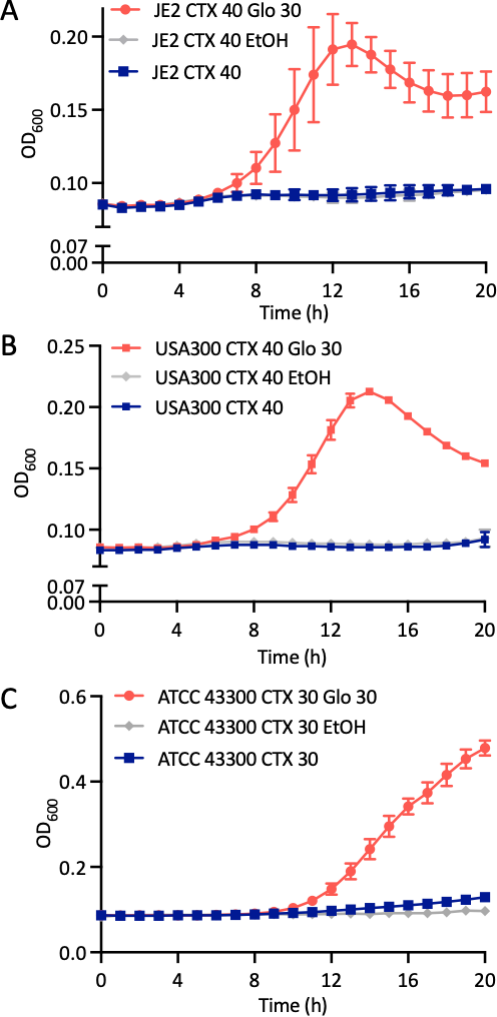
Globomycin increases cefotaxime resistance in JE2, FPR3757, and ATCC43300. (**A**) JE2 was grown in MHB 2% NaCl supplemented with cefotaxime (CTX) 40 µg/mL, MHB 2% NaCl supplemented with CTX 40 µg/mL and globomycin (Glo) 30 µg/mL, or MHB 2% NaCl supplemented with CTX 40 µg/mL and 0.6% ethanol (control solvent for Glo). (**B**) FPR3757 was grown in MHB 2% NaCl supplemented with CTX 40 µg/mL, MHB 2% NaCl supplemented with CTX 40 µg/mL and Glo 30 µg/mL, or MHB 2% NaCl supplemented with CTX 40 µg/mL and 0.6% ethanol (EtOH, control solvent for Glo). (**C**) ATCC 43300 was grown in MHB 2% NaCl supplemented with CTX 30 µg/mL, MHB 2% NaCl supplemented with CTX 30 µg/mL and Glo 30 µg/mL, or MHB 2% NaCl supplemented with CTX 30 µg/mL and 0.6% ethanol (control solvent for Glo). The CTX and Glo concentrations used in these experiments were determined empirically for each strain. The cultures were grown in a Tecan Sunrise incubated microplate reader for 20 h at 35°C OD_600_ was recorded at 1 h intervals and growth curves were plotted in Prism software (GraphPad). The data presented are the average of three independent biological replicates, and error bars represent standard deviations.

To determine if globomycin could also increase resistance to other classes of β-lactam antibiotics, its effect on cefotaxime resistance in JE2, FPR3757, and ATCC43300 was evaluated. Cefotaxime was chosen because it is a third-generation cephalosporin with broad spectrum activity against Gram-positive and Gram-negative bacteria commonly used in the clinic, whereas oxacillin is a narrow-spectrum penicillinase-resistant β-lactam. Cefotaxime 40 µg/mL inhibited the growth of JE2 and FPR3757 (Fig. 4A and B), while 30 µg/mL inhibited the growth of ATCC43300 (Fig. 4C). Globomycin (30 µg/mL) rescued the growth of all three strains in cefotaxime (Fig. 4A through C), suggesting that globomycin-mediated inhibition of LspA may broadly antagonize the activity of β-lactam antibiotics.

### Mutation of *lgt* in the *lspA* background restores wild-type levels of β-lactam resistance

LspA catalyzes the second major step in the lipoprotein processing pathway. To probe the contribution of lipoprotein processing to LspA-controlled oxacillin resistance, we compared the impact of *lgt, lspA,* and *lgt/lspA* mutations on growth and resistance to oxacillin, as well as cefotaxime, nafcillin, and vancomycin. Lgt catalyzes the addition of a diacylglycerol moiety onto preprolipoproteins, from which the signal peptide is then cleaved by LspA. To construct a *lspA/lgt* double mutant, the erythromycin resistance marker of the *lspA*::Tn allele in NE1757 was first exchanged for a markerless transposon to generate a strain designated *lspA* MM into which the erythromycin-marked *lgt*::Tn allele from NE1905 was transduced. All strains were confirmed using comparative WGS analysis.

**Fig 5:**
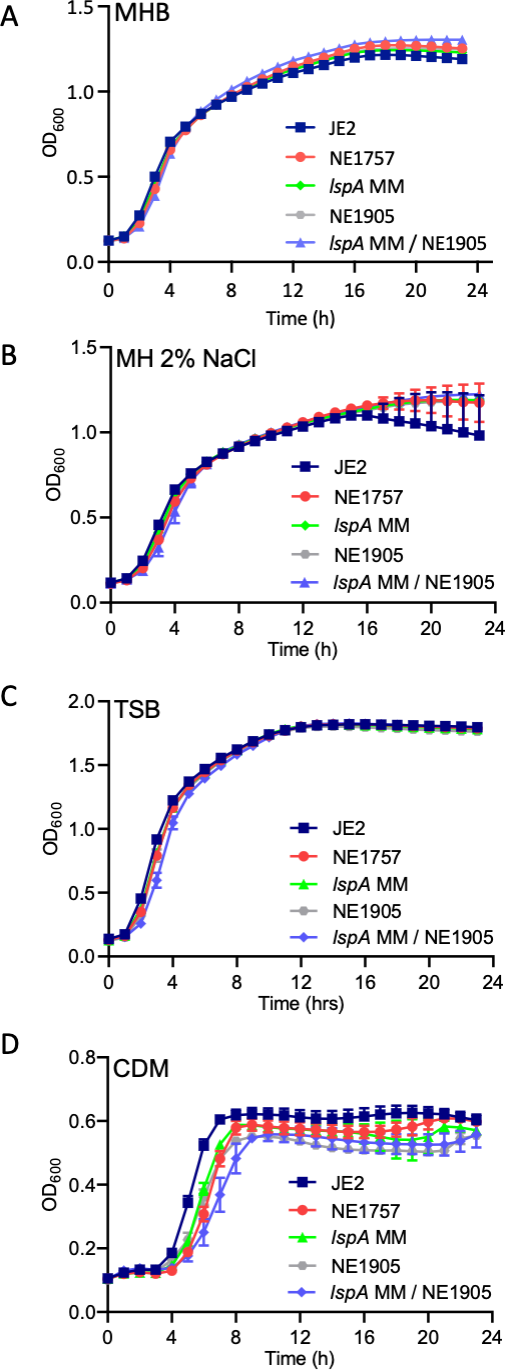
Mutation of *lspA* or *lgt* impacts growth in nutrient-limited media but not in complex media. Growth of JE2 wild type, NE1757 (*lspA*::Tn), *lspA* MM (markerless *lspA*::Tn), NE1905 (*lgt*::Tn), and *lspA* MM/NE1905 (*lspA*::Tn MM / *lgt*::Tn) in Mueller-Hinton broth (**A**), Mueller-Hinton broth with 2% NaCl (**B**), Tryptic Soya broth (**C**), and chemically-defined medium (**D**). Growth experiments were performed in 96-well hydrophobic plates in a Tecan Sunrise incubated microplate reader for 24 h at 37°C. OD_600_ was recorded at 1 h intervals, and growth curves were plotted in Prism software (GraphPad). The data presented are the average of three independent biological replicates, and error bars represent standard deviations.

Comparison of growth of JE2, NE1757, *lspA* MM, NE1905, and the *lspA/lgt* double mutant *lspA* MM /NE1905 revealed no significant differences in MHB, MHB with 2% NaCl, or TSB (Fig. 5A through C). However, consistent with the previous analysis of a *lgt* mutant (40), the *lspA, lgt,* and, in particular, the *lspA*/*lgt* mutants exhibited impaired growth in chemically defined medium (CDM) (Fig. 5D), suggesting that aberrant lipoprotein processing may affect nutrient acquisition under substrate-limiting conditions.

The *lgt* mutant NE1905 exhibited no changes in susceptibility to oxacillin, cefotaxime, nafcillin, or vancomycin (Table 1). As observed for oxacillin, the *lspA* mutant NE1757 was more resistant to cefotaxime and nafcillin, and these phenotypes were complemented by the p*lspA* plasmid (Table 1). Neither the *lspA* nor *lgt* mutations increased resistance to vancomycin (Table 1). Oxacillin and nafcillin MICs were reduced to wild-type levels in the *lspA*/*lgt* double mutant, and the cefotaxime MIC was reduced from 256 to 128 μg/mL (Table 1).

**Fig 6:**
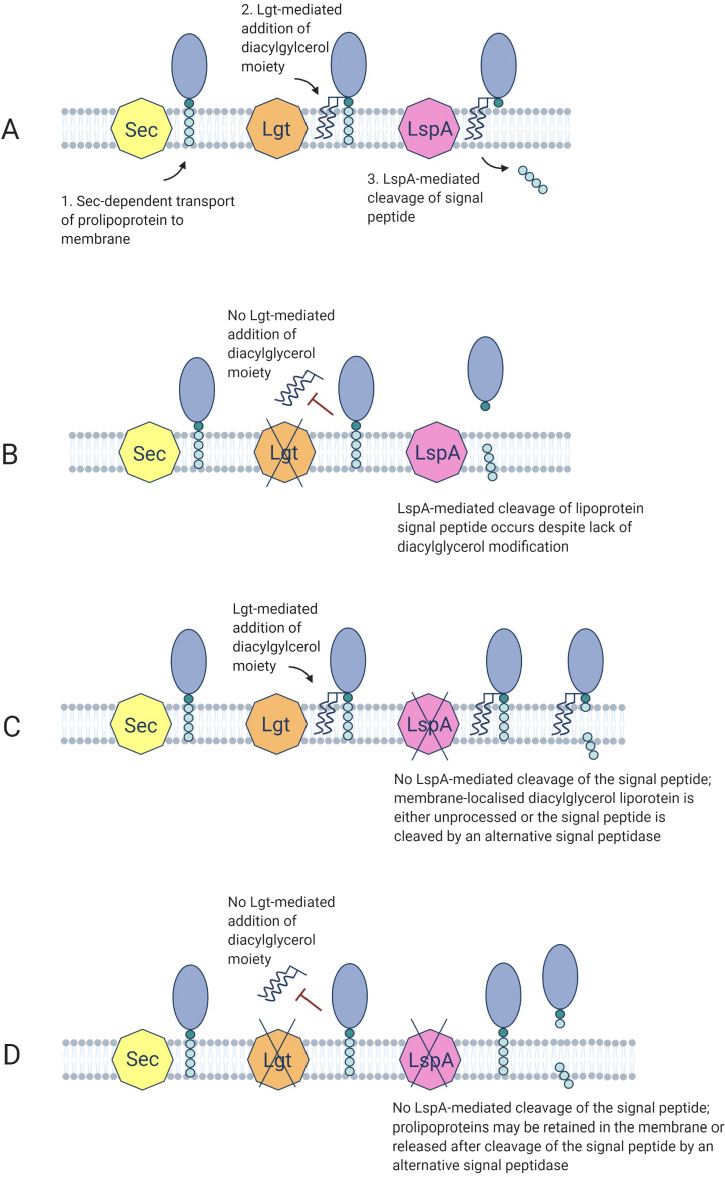
Suggested model depicting the possible impacts of *lgt*, *lspA,* and *lgt/lspA* mutations on lipoprotein processing in *S. aureus*. (**A**) Overview of lipoprotein processing in Gram-positive bacteria. (**B**) The *lgt* mutant lacks diacylglyceryl transferase activity potentially blocking accumulation of the acylated prolipoprotein. In this scenario, the prolipoprotein may be released from the membrane following LspA-mediated cleavage of the signal peptide. (**C**) In the *lspA* mutant, LspA-mediated cleavage of the signal peptide is proposed to be lost, but an alternative signal peptidase may undertake this activity. (**D**) We propose that prolipoproteins are not processed in mutants lacking Lgt and LspA activity and may be retained in the membrane or released after cleavage of the signal peptide by an alternative signal peptidase. Illustrations created with Biorender.com.

Taken together, these data indicate that while mutation of *lspA* or *lgt* or both impact growth in CDM, only mutation of *lspA* alone is associated with increased β-lactam resistance. The *lgt* mutation and possible accumulation of unprocessed prolipoproteins (Fig. 6A and B) does not increase β-lactam resistance, whereas the possible accumulation of diacylglyceryl-lipoprotein in a *lspA* mutant (Fig. 6C and D) is associated with this phenotype. The oxacillin, cefotaxime, and nafcillin MICs of the *ecsB* mutant from the NTML were the same as wild type (Table 1) indicating that downstream processing of the LspA-cleaved signal peptide is not associated with altered β-lactam resistance.

## DISCUSSION

Advances in our understanding of the accessory factors that control levels of *mecA*/PBP2a-dependent resistance to methicillin have the potential to reveal new therapeutic targets and drugs that may facilitate the reintroduction of other β-lactam antibiotics for the treatment of MRSA infections. In this study, we demonstrated that mutation of the lipoprotein processing pathway gene *lspA* or inhibition of LspA with globomycin increased resistance to β-lactam antibiotics. Although numerous mutations impacting the stringent response (ppGpp) and c-di-AMP signaling are associated with the transition from a heterogeneous to homogeneous pattern of resistance and elevated PBP2a expression (7, 8), our data show that the *lspA* mutation was not associated with a HoR phenotype or increased PBP2a expression. On the other hand, changes in β-lactam resistance independent of altered PBP2a regulation has long been known (52–57), and several auxiliary factors known to influence β-lactam resistance in MRSA have been described (54, 58–62). In addition to unchanged PBP2a protein levels, no evidence of peptidoglycan remodeling was observed in NE1757 compared to JE2 after the growth in the presence or absence of oxacillin, potentially implicating wall teichoic acid (WTA) or lipoteichoic acid (LTA) synthesis or stability in the *lspA* mutant phenotype. One of the numerous lipoproteins processed by LspA is PrsA, a chaperone and foldase protein, implicated in proper PBP2a folding and β-lactam resistance (63–65). Although it is difficult to reconcile increased β-lactam susceptibility in mutants lacking *prsA* (63–65) with increased β-lactam resistance in the *lspA* mutant, the possibility that PBP2a folding/activity is increased in a PrsA-dependent manner cannot be ruled out. Inhibition of WTA synthesis was previously shown to decrease β-lactam resistance in a PBP2a-independent manner (58). Reduced LTA stability was recently correlated with a PBP2a-independent reduction in β-lactam resistance in auxiliary factor *auxA* and *auxB* mutants (54). Interestingly, AuxA is structurally similar to SecDF (54) and may interact with the Sec pathway and lipoprotein processing (Fig. 6).

Consistent with previous studies of lipoprotein pathway processing mutants in *S. aureus* (40), *Listeria monocytogenes* (66), and *Streptococcus agalactiae* (67), our analysis also showed that the *lgt, lspA,* and *lgt*/*lspA* double mutants all exhibited impaired growth in CDM but not in TSB or MHB indicating that the impact of lipoprotein processing pathway mutations on nutrient acquisition and growth under nutrient-limiting conditions can be compensated in rich media.

Mutation of the *lgt* gene from the lipoprotein processing pathway did not affect β-lactam resistance, and introduction of the *lgt* mutation into a *lspA* mutant was accompanied by a restoration of wild-type levels of resistance. These data suggest that accumulation of diacylglycerol lipoprotein may be associated with elevated β-lactam resistance. Consistent with this possibility, lipoproteins were retained in the membrane of a *S. agalactiae lspA* mutant but were released into the supernatant in large concentrations by *lgt* and *lgt/lspA* mutants (67). Lipoproteins synthesized by the *S. agalactiae lspA* mutant retained their signal peptide, which was absent in a *lgt* mutant with LspA activity (67). Importantly, signal peptide processing also occurred in the *lgt/lspA* double mutant, albeit with cleavage occurring between different amino acids, implicating the involvement of an alternative signal peptidase (67). Our analysis revealed no change in oxacillin susceptibility in the MRSA *lgt*/*lspA* double mutant, indicating that even if an alternative peptidase can cleave the signal peptide, this may not impact β-lactam resistance. Furthermore, mutation of *ecsB*, which has recently been implicated in export of linear peptides from the lipoprotein processing pathway in *S. aureus* (37), did not change the oxacillin MIC in JE2 (Table 1) also indicating that downstream processing of signal peptides cleaved from lipoproteins by LspA is not associated with altered β-lactam resistance. In *L. monocytogen*es, the deletion of *lgt* also led to significant release of lipoproteins into the supernatant. However, the treatment of the *L. monocytogenes lgt* mutant with globomycin (inhibiting LspA activity) resulted in enhanced lipoprotein retention in the membrane (66), suggesting that the impact of globomycin and *lspA* mutation on lipoprotein processing is not necessarily the same. In a *S. aureus lgt* mutant, the Götz group reported that the majority of lipoprotein (lacking signal peptide) was released into the supernatant (40).

Taken together, the data suggest that accumulation of membrane-anchored diacylglycerol lipoprotein with uncleaved signal peptide, or lipoprotein that is mislocalized or released due to aberrant signal peptide processing by an alternative peptidase, is accompanied by increased β-lactam resistance in MRSA.

## MATERIALS AND METHODS

### Bacterial strains and culture conditions

Bacterial strains and plasmids used in this study are listed in Table S1. *Escherichia coli* strains were grown in Luria-Bertani (LB) broth or agar (LBA). *S. aureus* strains were grown in Tryptic Soy Broth (TSB), Tryptic Soy Agar (TSA), or chemically defined medium (CDM) (68). Mueller-Hinton Broth (MHB) or Mueller-Hinton Agar (MHA) (Oxoid) supplemented with 2% NaCl where indicated, was used for antimicrobial susceptibility testing (AST). Antibiotic concentrations used were 10 µg/mL erythromycin, 10 µg/mL chloramphenicol, 75 µg/mL kanamycin, 100 µg/mL ampicillin.

Two hundred and fifty milliliter flasks were filled with 25 mL growth media, and overnight cultures were used to inoculate the media at a starting OD_600_ of 0.05. Overnight cultures were grown in TSB and washed once in 5 mL PBS before being used to inoculate CDM and chemically defined medium with glucose (CDMG) cultures.

Flasks were incubated at 37°C shaking at 200 rpm. OD_600_ readings were measured at 1–2 h intervals. Colony forming units (CFU) were enumerated in serially diluted 20 µL aliquots removed from flask cultures. Three independent biological replicates were performed for each strain, and the data were plotted using GraphPad Prism software.

Data from growth experiments in a Tecan Sunrise microplate instrument were recorded by Magellan software. Overnight cultures were adjusted to an OD_600_ of 1 in fresh media and 10 µL inoculated into 200 µL media per well before being incubated at 37°C for 24 h with shaking. OD_600_ was recorded every 15 min. For CDM, overnight TSB cultures were first washed in 5 mL PBS and adjusted to OD_600_ of 1 in PBS.

### Genetic manipulation of *S. aureus*

Phage 80α transduction was used to verify the association between antibiotic resistance phenotypes and transposon insertion-marked mutations from the NTML as described previously (45). Transductants were verified by PCR amplification of the target locus using primers listed in Table S2. The plasmid pTnT, which contains a truncated, markerless transposon was used to construct a markerless *lspA* mutant designated *lspA* MM, as described previously (69). A double *lspA*/*lgt* double mutant was subsequently constructed using phage 80α to transduce the *lgt*::Tn allele from NE1905 into *lspA* MM. A 1,324 bp fragment encompassing the *lspA* gene and its upstream putative promoter region was PCR amplified from JE2 genomic DNA using primers NE1757_INF#3_Fwd and NE1757_INF#3_Rev (Table S2), purified with GenElute PCR Clean-Up Kit and cloned into the *E. coli - Staphylococcus* shuttle vector pLI50 digested with *Eco*RI (New England Biolabs) using In-Fusion HD Cloning Kit (Clontech) to generate p*lspA*. Using electroporation*,* p*lspA* was transformed sequentially into *E. coli* HST08 (Clontech) *S. aureus* RN4220 and NE1757.

### Disk diffusion susceptibility assays

Cefoxitin disk diffusion susceptibility assays were performed in accordance with CLSI guidelines (70). Briefly, isolates were grown at 37°C on MHA for 24 h and 5–6 colonies were resuspended in 0.85% saline to OD_600_ of 0.08–0.1 (0.5 McFarland; 1 × 10^8^ CFU/mL) and swabbed onto MHA plates with a uniform agar depth of 4 mm. A 30 µg cefoxitin disk (Oxoid) was applied, the plate incubated at 35°C for 16–18 h, and the zone of inhibition diameter measured. Strains were classified as sensitive, intermediate, or resistant, according to CLSI criteria (71).

### Minimum inhibitory concentration measurements

For oxacillin M.I.C.Evaluators (Oxoid), 5–6 colonies from 24 h MHA plates were resuspended in 0.85% saline to OD_600_ of 0.08–0.1, (0.5 McFarland standard) and evenly swabbed onto MHA 2% NaCl (4 mm agar depth). An M.I.C.Evaluator strip was applied, and the plate was incubated at 35°C for 24 h. Three biological repeats were performed for each strain.

MIC measurements by broth microdilution or agar dilution were performed in accordance with CLSI methods for dilution susceptibility testing of staphylococci (72). For broth microdilution MIC measurements using 96-well plates, each plate row was used to prepare twofold dilutions of antibiotic in MHB, typically ranging from 256 to 0.5 µg/mL across 10 wells. For oxacillin and nafcillin MIC measurements, MHB containing 2% NaCl was used. 5-6 colonies from 24 h MHA plates were resuspended in 0.85% saline to OD_600_ of 0.08–0.1 (0.5 McFarland standard) and diluted 1:20 in 0.85% saline, and 10 µL of this cell suspension used to inoculate each well (approximately 5 × 10^4^ CFU/well) in a final volume of 100 mL. The plates were incubated at 35°C for 16–20 h or 24 h incubation for oxacillin and nafcillin. The MIC was the lowest concentration of antimicrobial agent that completely inhibited growth.

Freshly prepared MHA plates (with 2% NaCl when using oxacillin and nafcillin) were supplemented with antimicrobial agents at 0.5, 1, 2, 4, 8, 16, 32, 64, 128, and 256 µg/mL to perform agar dilution MIC measurements. For the inoculum, 5–6 colonies from a 24-h MHA plate were resuspended in 0.85% saline to give 0.5 McFarland or OD_600_ of 0.08–0.1 (1–2 × 10^8^ CFU/mL). This was further diluted 1:20 in 0.85% saline, and 4 µL was spot-inoculated onto each plate, yielding a final inoculum of 10^4^ CFU per spot of 5–8 mm diameter. The MIC was the lowest concentration of antimicrobial agent that completely inhibited growth after 16–18 h (24 h for oxacillin and nafcillin) at 35°C, disregarding a single colony or a faint haze associated with the inoculum. MIC results were interpreted using CLSI standard M100, and strains classified as susceptible or resistant (71).

### Autolytic activity assays

Two-hundred microliters from overnight cultures (20 mL) were inoculated into 20 mL TSB, grown at 37°C (200 rpm) to OD_600_ of 0.5, washed with 20 mL cold PBS, resuspended in 1 mL cold PBS, and finally adjusted to OD_600_ of 1. Triton X-100 was added at a final concentration of 0.1% (vol/vol), and the cell suspension was incubated at 37°C with shaking (200 rpm). OD_600_ was recorded every 30 min for 4 h. Autolytic activity was expressed as a percentage of the initial OD_600_. NE406 (*atl*::Tn) was used as a negative control, and at least three biological replicates were performed for each strain.

### Globomycin and β-lactam antibiotic synergy/antagonism assays

One hundred microliters of MHB cefotaxime or MHB 2% NaCl oxacillin was added to the individual wells of 96-well plates. The oxacillin or cefotaxime concentration chosen for each strain was based on approximate MICs, i.e., the lowest antibiotic concentration that inhibited growth. Overnight MHB cultures were resuspended in PBS at OD_600_ = 0.1 (0.5 McFarland standard) and then further diluted 1:20 before 10 µL (approximately 7.5 × 10^4^ CFU/mL) was added to each well, and the plates were incubated at 35°C with shaking on a Tecan Sunrise microplate instrument for 20 h (cefotaxime) or 24 h (oxacillin). Globomycin ranging from 10 to 100 µg/mL was added to the cefotaxime or oxacillin cultures to measure potential synergism or antagonism. Three independent biological replicates were performed for each strain and antibiotic combination.

### PBP2a western blot analysis

Overnight MHB cultures were used to inoculate 25 mL of MHB 2% NaCl, with or without 0.5 µg/mL oxacillin to a starting OD_600_ of 0.05, incubated at 35°C (200 rpm shaking) until an OD_600_ of 0.8 was reached before the cells were pelleted at 21,000 × *g* and resuspended in PBS to an OD_600_ of 10. Six microliters of lysostaphin (AMBI, USA) (10 µg/mL) and 1 µL of DNase (Sigma) (10 µg/mL) were added to 500 µL of this concentrated cell suspension before being incubated at 37°C for 40 min. Next, 50 µL of 10% SDS was added and the incubation continued for a further 20 min. The lysed cells were then pelleted in a microcentrifuge for 15 min (21,000 × *g*), following which the protein-containing supernatant was collected and total protein concentration determined using the Pierce BCA Protein Assay Kit. Samples containing 8 µg total protein were mixed 1:1 with protein loading buffer (2×) (National Diagnostics) and incubated at 95°C for 5 min and loaded onto a 7.5% Tris-Glycine gel and separated at 120 V for 60 min. Electrophoretic transfer to a PVDF membrane was carried out at 30 V for 30 min on the Trans-Blot Turbo Transfer System (Biorad). The PVDF membrane was blocked overnight in 5% skim milk powder in PBS at 4°C. The following day, the membrane was washed in fresh PBS. Anti-PBP2a (Abnova) was diluted 1:1,000 in PBS-Tween 20 (0.1%) and incubated with the membrane for 1 h at room temperature. The membrane was washed in PBS to remove unbound antibody. The secondary antibody, HRP-rec-Protein G (Invitrogen) was diluted 1:2,000 in PBS-Tween 20 (0.1%) and incubated with the membrane at room temperature for 1 h. Chemiluminescence detection was performed with the Amersham ECL Western Blotting Detection kit. The blot was imaged using a LICOR C-Digit Blot scanner using the Image Studio Software (version 6.1). Four independent experiments were performed, and representative images of the developed PVDF membranes were recorded.

### Population level antibiotic resistance profile analysis

Characterization of the population resistance profile was performed as described previously (73). Overnight cultures were grown in TSB, adjusted to an OD_600_ of 1, 10-fold serially diluted from 10^−1^ to 10^−7^, and a 20 µL aliquot of each dilution plated onto a series of TSA agar plates supplemented with oxacillin 0.25, 0.5, 1, 2, 4, 8, 16, 32, 64, and 128 µg/mL. CFUs were enumerated after overnight incubation at 37°C, and the results were expressed as colony forming units per mL (CFU/mL) at each oxacillin concentration. Three independent experiments were performed for each strain.

### Antibiotic tolerance assay

Tolerance assays were performed as described previously (74). Briefly overnight TSB cultures were sub-cultured into 25 mL of fresh TSB in 250 mL flasks at a starting OD_600_ of 0.05 and grown to an OD_600_ of 0.5 at 37°C with 200 rpm shaking. At this time (*T*_0_), an aliquot was removed for CFU enumeration and 12.5 µg/mL oxacillin promptly added before the cultures were re-incubated. Antibiotic tolerance was expressed as the % CFU/mL after 2, 4, 6, 8, and 24 h growth in the antibiotic compared to the CFU/mL at *T*_0_. The results represent three biological replicates of each strain.

### Genomic DNA extraction and whole-genome sequencing

Genomic DNA (gDNA) extractions were performed as previously described (75) using the Wizard Genomic DNA Purification Kit (Promega) following pre-treatment of *S. aureus* cells with 10 µg/mL lysostaphin (Ambi Products LLC) at 37°C for 30 min. The genome sequencing for NE1757 (*lspA*::Tn) was performed by MicrobesNG using an Illumina sequencing platform with 2 × 250 bp paired-end reads. DNA libraries for *lspA* MM, NE1905 (*lgt::tn*), and *lspA* MM/*lgt* double mutant were prepared using an Illumina Nextera XT DNA Library Prep kit, validating size distribution by gel electrophoresis, and bead-normalizing the libraries. An Illumina MiSeq v2 600 cycle kit was used for genome sequencing, generating 300-bp paired end reads. PhiX was used as a sequencer loading control. CLC Genomics Workbench software (Version 20) (Qiagen) was used for genome sequencing analysis of strains. As a reference genome, a contig was produced for wild-type JE2 (from project PRJEB59981 [57]) by mapping Illumina reads onto the closely related FPR3757 genome sequence (RefSeq accession number NC_07793.1). The Illumina short read sequences from NE1757 were then mapped onto the assembled JE2 sequence and the presence of the transposon insertion confirmed. Single nucleotide polymorphisms (SNPs), deletions, or insertions were mapped in the NE1757 genome and the presence of large deletions ruled out by manually searching for zero coverage regions using the CLC Genomics Workbench software as previously described (76).

### Peptidoglycan analysis

For each strain and growth condition tested, independent quadruplicate 50 mL cultures were grown to an OD_600_ of 0.5 and harvested and resuspended in 5 mL PBS before peptidoglycan was extracted as described previously (45, 77). Mass spectrometry was performed on a Waters XevoG2-XS QTof mass spectrometer. Structural characterization of muropeptides was determined based on their MS data and MS/MS fragmentation pattern, matched with PG composition and structure reported previously (78–81).

## Data Availability

Whole-genome sequence data are available from the European Nucleotide Archive (https://www.ebi.ac.uk/ena), Registered Project PRJEB96174, sample accession numbers ERS25834860–ERS25834863 (https://www.ebi.ac.uk/ena/browser/view/PRJEB96174). The SAUSA300_FRP3757 (TaxID: 451515) reference genome sequence is available from NCBI (www.ncbi.nlm.nih.gov).

## SUPPLEMENTAL MATERIAL

Fig. S1 aac.01276-25-s0001.pdf Supplemental figure 1.

Fig. S2 aac.01276-25-s0002.pdf Supplemental figure 2.

Fig. S3 aac.01276-25-s0003.pdf Supplemental figure 3.

Fig. S4 aac.01276-25-s0004.pdf Supplemental figure 4.

Fig. S5 aac.01276-25-s0005.pdf Supplemental figure 5.

Fig. S6 aac.01276-25-s0006.pdf Supplemental figure 6.

Table S1 aac.01276-25-s0007.docx Bacterial strains and plasmids used in this study.

Table S2 aac.01276-25-s0008.docx Oligonucleotides used in this study.

ASM does not own the copyrights to Supplemental Material that may be linked to, or accessed through, an article. The authors have granted ASM a non-exclusive, world-wide license to publish the Supplemental Material files. Please contact the corresponding author directly for reuse.
