# Supplementary material for: Mutation of lipoprotein processing pathway gene lspA or inhibition of LspA activity by globomycin increases MRSA resistance to β-lactam antibiotics
Source: Antimicrob Agents Chemother. 2025 Dec 29;70(2):e01276-25. doi: 10.1128/aac.01276-25 (PMC12888878; doi:10.1128/aac.01276-25)
Supplement: Fig. S1 — Supplemental figure 1. [file aac.01276-25-s0001.pdf]

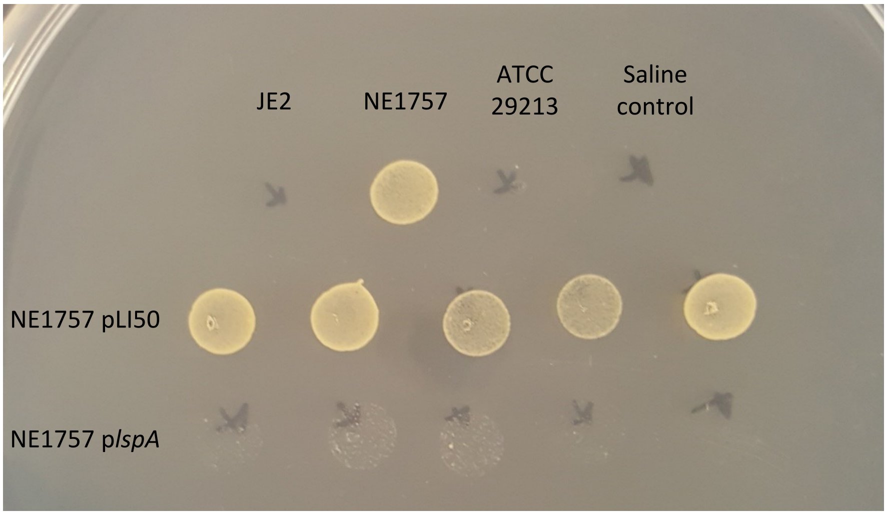

Supplementary Fig. S1. Complementation of the NE1757 mutant with the *lspA* gene is accompanied by a wild type oxacillin resistance phenotype. JE2, NE1757 (*lspA*::Tn), NE1757 pLI50 and NE1757 p*lspA* were grown on MHA 2% NaCl supplemented with oxacillin 32 µg/ml. The plate shows 5 biological replicates each for NE1757 pLI50 and NE1757 p*lspA.* ATCC 29213 was included as an oxacillin susceptible control.
