# Supplementary material for: Mutation of lipoprotein processing pathway gene lspA or inhibition of LspA activity by globomycin increases MRSA resistance to β-lactam antibiotics
Source: Antimicrob Agents Chemother. 2025 Dec 29;70(2):e01276-25. doi: 10.1128/aac.01276-25 (PMC12888878; doi:10.1128/aac.01276-25)
Supplement: Fig. S2 — Supplemental figure 2. [file aac.01276-25-s0002.pdf]

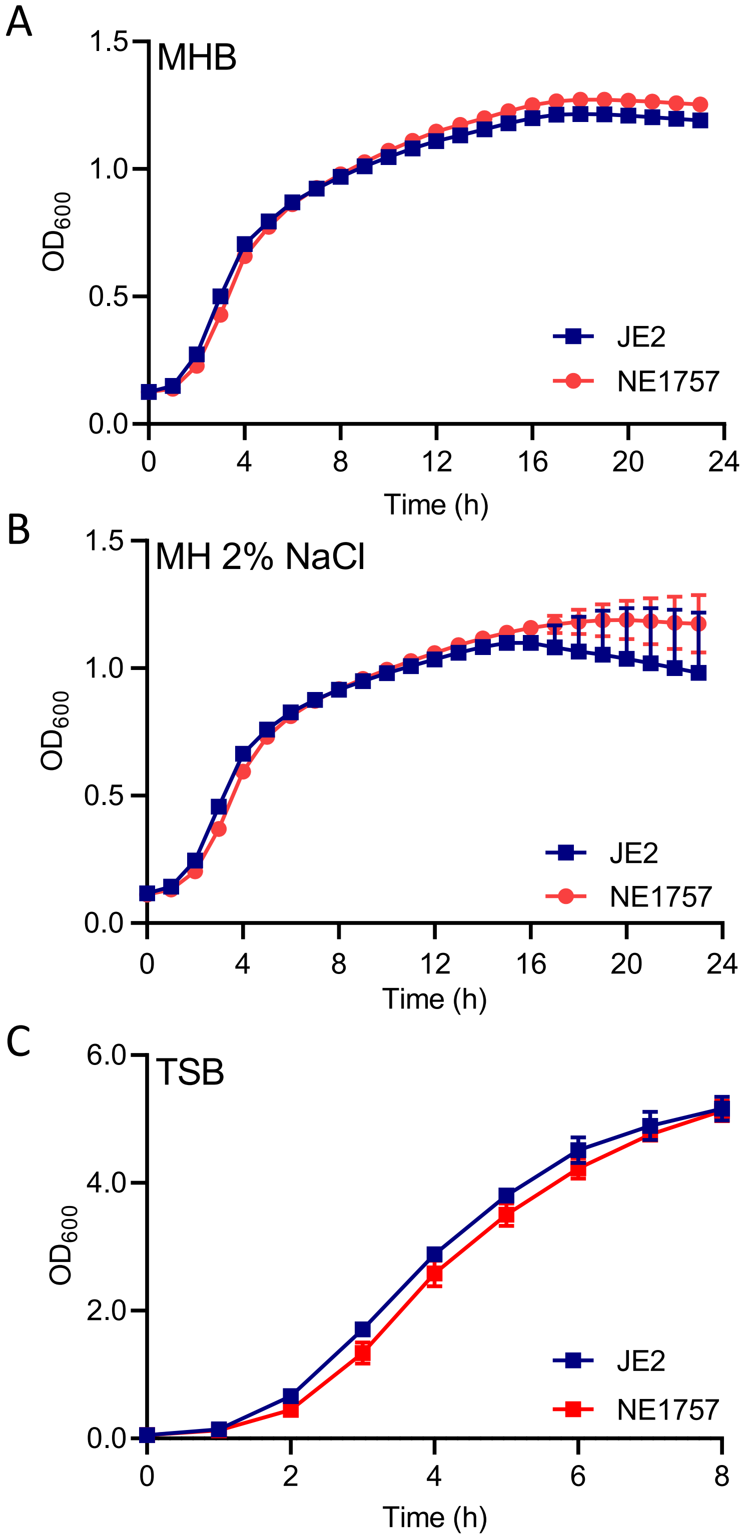

Supplementary Fig. S2. Mutation of *lspA* does not impact growth in MHB or TSB media. Growth of JE2 and NE1757 cultures in **(A)** MHB, **(B)** MHB 2% NaCl and **(C)** TSB. MHB cultures were grown in 96-well plates in a Tecan Sunrise incubated microplate reader for 24 h at 37°C. The OD_{600} was recorded at 15 min intervals and growth curves were plotted in Prism software (GraphPad). TSB cultures were grown in flasks and the OD_{600} monitored every 2 h. All data presented are the average of 3 independent biological replicates, and error bars represent standard deviations.
