# Supplementary material for: Mutation of lipoprotein processing pathway gene lspA or inhibition of LspA activity by globomycin increases MRSA resistance to β-lactam antibiotics
Source: Antimicrob Agents Chemother. 2025 Dec 29;70(2):e01276-25. doi: 10.1128/aac.01276-25 (PMC12888878; doi:10.1128/aac.01276-25)
Supplement: Fig. S3 — Supplemental figure 3. [file aac.01276-25-s0003.pdf]

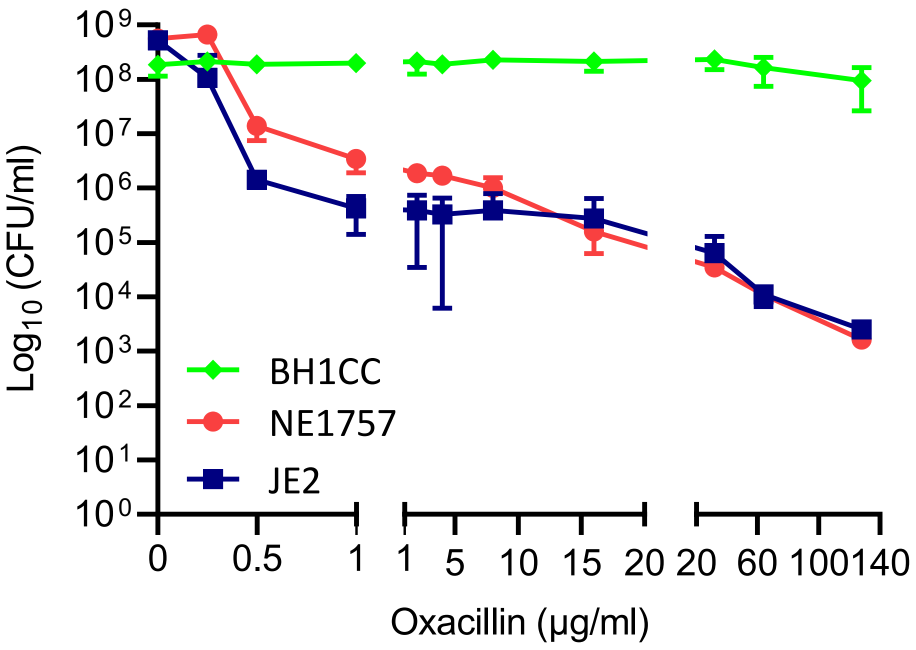

Supplementary Fig. S3. NE1757 (*lspA*) exhibits heterogenous resistance to oxacillin. Overnight cultures of JE2, NE1757 and BH1CC were grown in TSB, adjusted to OD_{600} of 1, serially diluted and plated onto TSA and TSA supplemented with 0.25, 0.5, 1, 2, 4, 8, 16, 32, 64 and 128 µg/ml oxacillin. CFUs were enumerated after overnight incubation at 37°C. The data are expressed as CFU/ml at each oxacillin concentration, plotted using Prism software (GraphPad). Three independent population analysis profile experiments were performed, and error bars represent standard deviations. BH1CC, which exhibits homogenous oxacillin resistance, was included as a control.
