# Supplementary material for: Mutation of lipoprotein processing pathway gene lspA or inhibition of LspA activity by globomycin increases MRSA resistance to β-lactam antibiotics
Source: Antimicrob Agents Chemother. 2025 Dec 29;70(2):e01276-25. doi: 10.1128/aac.01276-25 (PMC12888878; doi:10.1128/aac.01276-25)
Supplement: Fig. S4 — Supplemental figure 4. [file aac.01276-25-s0004.pdf]

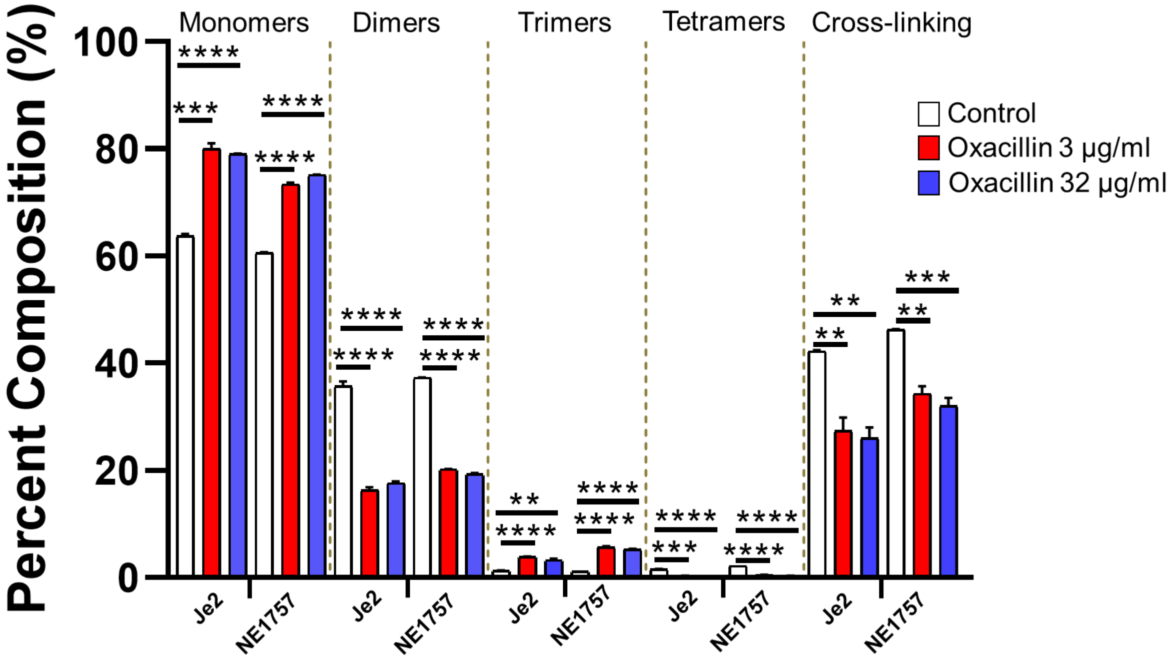


**Supplementary Fig. S4.** Relative proportions of cell wall muropeptide fractions based on oligomerization and relative cross-linking efficiency of cell wall muropeptide fractions in peptidoglycan extracted from JE2 and NE1757. Cells were collected from cultures grown to exponential phase in MHB or MHB supplemented with oxacillin 3 µg/ml or 32 µg/ml. Each profile shown is a representative of 3 biological replicates. Significant differences determined using Students t-test (**P < .01; ***P < .001; ****P<.0001).
