# Supplementary material for: Mutation of lipoprotein processing pathway gene lspA or inhibition of LspA activity by globomycin increases MRSA resistance to β-lactam antibiotics
Source: Antimicrob Agents Chemother. 2025 Dec 29;70(2):e01276-25. doi: 10.1128/aac.01276-25 (PMC12888878; doi:10.1128/aac.01276-25)
Supplement: Fig. S5 — Supplemental figure 5. [file aac.01276-25-s0005.pdf]

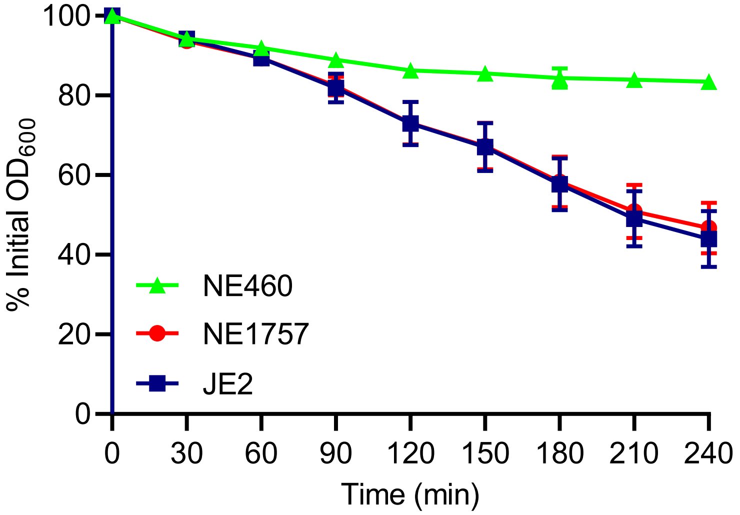

Supplementary Fig. S5. Autolytic activity is unaffected by the *lspA* mutation. Triton X-100-induced autolysis of JE2, NE1757 (*lspA::*Tn) and NE406 (*atl*::Tn, negative control). The strains were grown to OD_{600} = 0.5 in MHB medium at 37°C, before being washed in cold PBS and resuspended in 0.1% Triton X-100. The OD_{600} was monitored, and autolysis was expressed as a percentage of the initial OD_{600}. The experiments were repeated 3 independent times, plotted using Prism software (GraphPad) and standard deviations are shown.
