# Supplementary material for: Mutation of lipoprotein processing pathway gene lspA or inhibition of LspA activity by globomycin increases MRSA resistance to β-lactam antibiotics
Source: Antimicrob Agents Chemother. 2025 Dec 29;70(2):e01276-25. doi: 10.1128/aac.01276-25 (PMC12888878; doi:10.1128/aac.01276-25)
Supplement: Fig. S6 — Supplemental figure 6. [file aac.01276-25-s0006.pdf]

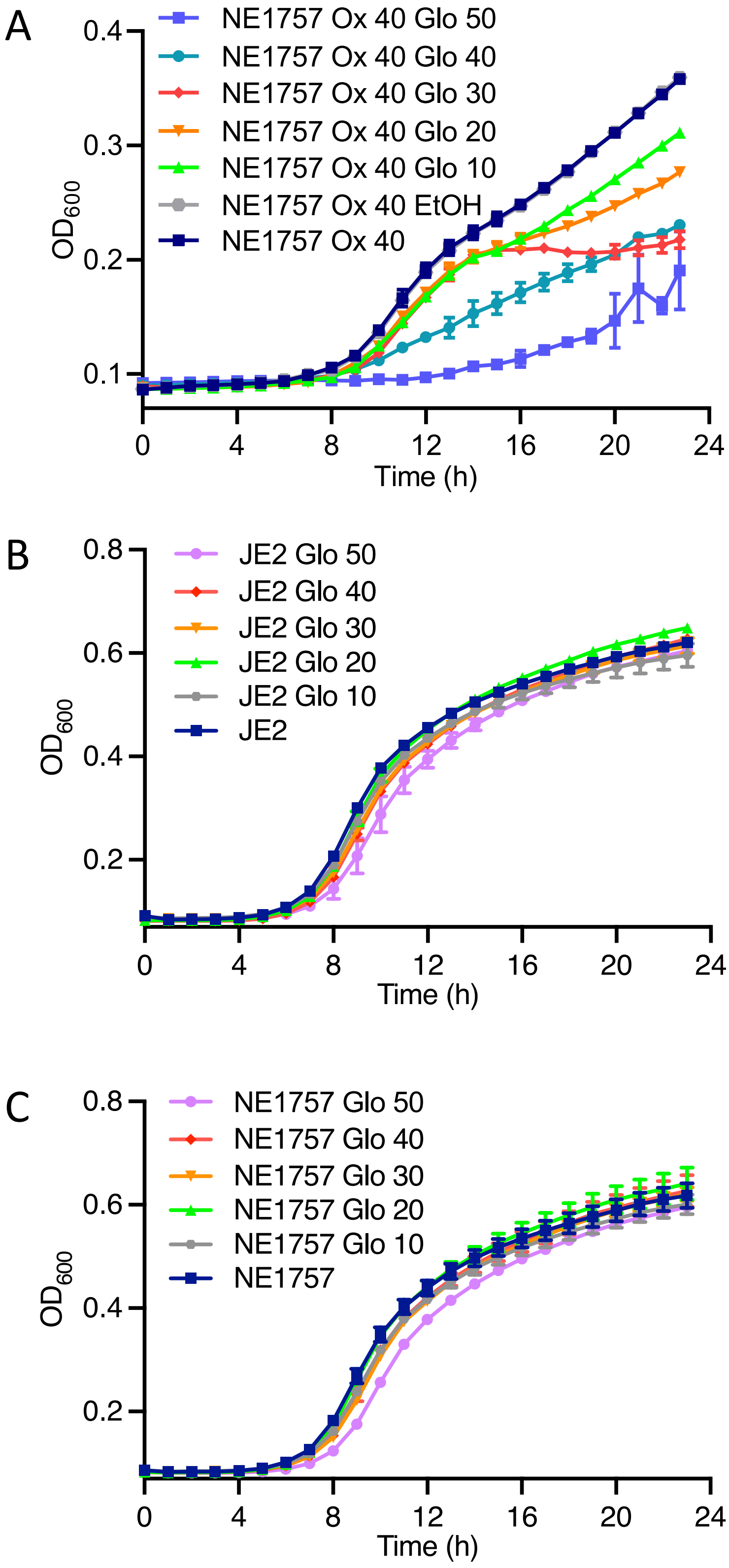


**Supplementary Fig. S6. A.** Globomycin does not increase oxacillin resistance in the *lspA* mutant NE1757. NE1757 was grown in MHB 2% NaCl supplemented with a sub-inhibitory concentration of oxacillin (40 µg/ml) and a range (10 – 50 µg/ml) of globomycin concentrations. The solvent for globomycin, 0.6% ethanol, was included as a control. **B and C.** JE2 (A) and NE1757 (B) were grown for 24 h in MHB supplemented with globomycin concentrations ranging from 10-50 µg/ml or MHB alone. The cultures were grown in a Tecan Sunrise incubated microplate reader for 24 h at 35°C. $OD_{600}$ was recorded at 15 min intervals and growth curves were plotted in Prism software (GraphPad). The data presented are the average of 3 independent biological replicates, and error bars represent standard deviations.
