# Supplementary material for: Mutation of lipoprotein processing pathway gene lspA or inhibition of LspA activity by globomycin increases MRSA resistance to β-lactam antibiotics
Source: Antimicrob Agents Chemother. 2025 Dec 29;70(2):e01276-25. doi: 10.1128/aac.01276-25 (PMC12888878; doi:10.1128/aac.01276-25)
Supplement: Table S1 — Bacterial strains and plasmids used in this study. [file aac.01276-25-s0007.docx]

**Supplementary Table S1.** Bacterial strains and plasmids used in this study

| **Strain/plasmid** | **Description and resistance** | | **Source/reference** |
| --- | --- | --- | --- |
| ***Escherichia coli* strains** | | |  |
| HST08 | TaKaRa *E. coli* HST08 Premium Electro-Cells | | TaKaRa |
| HST08 pLI50 | *E. coli* HST08 pLI50; Amp^R^ | | This study |
| HST08 p*lspA* | *E. coli* HST08 p*lspA*; Amp^R^ | | This study |
| ***Staphylococcus aureus* strains** | | |  |
| JE2 | JE2 (plasmid-cured derivative of strain LAC) | | (Fey *et al.*, 2013) |
| 8325-4 | NCTC 8325 derivative cured of prophages, methicillin susceptible, CC8. | | (Horsburgh *et al.*, 2002) |
| RN4220 | RN4220 (Restriction-deficient derivative of 8325-4) | | (Kreiswirth *et al.*, 1983) |
| BHICC | MRSA clinical isolate; SCC*mec* type II; CC8 | | (Gallagher *et al.*, 2020) |
| ATCC 29213 | Quality control strain for susceptibility testing; oxacillin sensitive | | *S. aureus* ATCC [29213](https://www.lgcstandards-atcc.org/products/all/25922.aspx) |
| ATCC 43300 | Quality control strain for susceptibility testing; oxacillin resistant, SCC*mec* Type II | | *S. aureus* ATCC [43300](https://www.lgcstandards-atcc.org/products/all/25922.aspx) |
| USA300 | USA300_FPR3757 | | (Diep *et al.*, 2006) |
| NE1757 (*lspA*) | JE2 *lspA*::Tn; Erm^R^ | | (Fey *et al.*, 2013) |
| NE1905 (*lgt*) | JE2 *lgt*::Tn; Erm^R^ | | (Fey *et al.*, 2013) |
| NE1757 MM | JE2 *lspA*:Tn (truncated, markerless Tn); Erm^S^ | | This study |
| NE1757/NE1905 | JE2 *lspA* MM *lgt*::Tn; Erm^R^ | | This study |
| RN4220 pTnT | RN4220 pTnT | | (Fey *et al.*, 2013) |
| RN4220 pLI50 | RN4220 pLI50; Cam^R^ | | This study |
| RN4220 p*lspA* | RN4220 p*lspA* | | This study |
| NE1757 p*lspA* | JE2 *lspA*::Tn p*lspA*; Erm^R^ Cam^R^ | | This study |
| NE1757 pLI50 | JE2 *lspA*::Tn pLI50; Erm^R^ Cam^R^ | | This study |
| JE2 pLI50 | JE2 pLI50; Cam^R^ | | This study |
| JE2 *lspA*::Tn #2 | JE2 *lspA*::Tn; Erm^R^ | | This study |
| JE2 *lspA*::Tn #4 | JE2 *lspA*::Tn; Erm^R^ | | This study |
| NE788 | JE2 *trkA*::Tn; Erm^R^ | | (Fey *et al.*, 2013) |
| NE460 | JE2 *atl*::Tn; Erm^R^ | | (Fey *et al.*, 2013) |
| NE107 | JE2 *ecsB*::Tn; Erm^r^ | | (Fey *et al.*, 2013) |
| **Plasmids** |  | |  |
| pTnT | Plasmid to replace Erm^R^ marker from transposon mutants with a markerless mutation. Cam^R^ | | (Bose *et al.*, 2013) |
| pLI50 | | *S. aureus*-*E. coli* shuttle vector | (Lee *et al.*, 1991) |
| p*lspA* | pLI50 carrying the *lspA* gene from JE2 | | This study |

References

Bose JL, Fey PD & Bayles KW (2013) Genetic tools to enhance the study of gene function and regulation in Staphylococcus aureus. *Appl Environ Microbiol* **79**: 2218-2224.

Diep BA, Gill SR, Chang RF*, et al.* (2006) Complete genome sequence of USA300, an epidemic clone of community-acquired meticillin-resistant Staphylococcus aureus. *Lancet* **367**: 731-739.

Fey PD, Endres JL, Yajjala VK, Widhelm TJ, Boissy RJ, Bose JL & Bayles KW (2013) A genetic resource for rapid and comprehensive phenotype screening of nonessential Staphylococcus aureus genes. *MBio* **4**: e00537-00512.

Gallagher LA, Shears RK, Fingleton C*, et al.* (2020) Impaired Alanine Transport or Exposure to d-Cycloserine Increases the Susceptibility of MRSA to beta-lactam Antibiotics. *J Infect Dis* **221**: 1000-1016.

Horsburgh MJ, Aish JL, White IJ, Shaw L, Lithgow JK & Foster SJ (2002) sigmaB modulates virulence determinant expression and stress resistance: characterization of a functional rsbU strain derived from Staphylococcus aureus 8325-4. *J Bacteriol* **184**: 5457-5467.

Kreiswirth BN, Lofdahl S, Betley MJ, O'Reilly M, Schlievert PM, Bergdoll MS & Novick RP (1983) The toxic shock syndrome exotoxin structural gene is not detectably transmitted by a prophage. *Nature* **305**: 709-712.

Lee CY, Buranen SL & Ye ZH (1991) Construction of single-copy integration vectors for Staphylococcus aureus. *Gene* **103**: 101-105.
