# Supplementary material for: Mutation of lipoprotein processing pathway gene lspA or inhibition of LspA activity by globomycin increases MRSA resistance to β-lactam antibiotics
Source: Antimicrob Agents Chemother. 2025 Dec 29;70(2):e01276-25. doi: 10.1128/aac.01276-25 (PMC12888878; doi:10.1128/aac.01276-25)
Supplement: Table S2 — Oligonucleotides used in this study. [file aac.01276-25-s0008.docx]

**Supplementary Table S2**. Oligonucleotides used in this study

| **Target** | **Name** | **Primer Sequence (5’ – 3’)** |
| --- | --- | --- |
| *lspA* (infusion primers) | NE1757_INF#3_Fwd | TCGTCTTCAAGAATTTTATGAAGGAGGCTGGGACA |
|  | NE1757_INF#3_Rev | TACCGAGCTCGAATTCAGGCAGCAACTTATCTACACG |
| **Tn-check primers** | **Name** | **Primer Sequence (5’ – 3’)** |
| *lspA* | NE1757_fwd | GTTCCAGCCTGCTTTCCTAATT |
|  | NE1757_rev | ACACGCATACCTGTTTGTTCT |
| *lgt* | NE1905_fwd | GCATTAACACGGCCGAAGAA |
|  | NE1905_rev | CAACCGTACCAGCTGCAAC |
